# Perspectives of Ferroelectric Wurtzite AlScN: Material Characteristics, Preparation, and Applications in Advanced Memory Devices

**DOI:** 10.3390/nano14110986

**Published:** 2024-06-06

**Authors:** Haiming Qin, Nan He, Cong Han, Miaocheng Zhang, Yu Wang, Rui Hu, Jiawen Wu, Weijing Shao, Mohamed Saadi, Hao Zhang, Youde Hu, Yi Liu, Xinpeng Wang, Yi Tong

**Affiliations:** 1 College of Integrated Circuit Science and Engineering, Nanjing University of Posts and Telecommunications, Nanjing 210023, China; 2023221108@njupt.edu.cn (H.Q.); hancong2023@gusulab.ac.cn (C.H.); liuyi@njupt.edu.cn (Y.L.); 2 Gusu Laboratory of Materials, 388 Ruoshui Road, Suzhou 215123, China; henan2021@gusulab.ac.cn (N.H.); zhangmiaocheng2021@gusulab.ac.cn (M.Z.); 2020020114@njupt.edu.cn (Y.W.); shaoweijing2020@gusulab.ac.cn (W.S.); saadi-mohamed@live.fr (M.S.); zhanghao2021@gusulab.ac.cn (H.Z.); huyoude2021@gusulab.ac.cn (Y.H.); 3 College of Electronic and Optical Engineering & College of Flexible Electronics (Future Technology), Nanjing University of Posts and Telecommunications, 9 Wenyuan Road, Nanjing 210023, China; 4 State Key Laboratory of Millimeter Waves, Southeast University, Nanjing 210096, China; 230239432@seu.edu.cn; 5 Institute of Functional Nano & Soft Materials, Soochow University, Suzhou 215123, China; jwwujwwu@stu.suda.edu.cn; 6 The Institute of Semiconductors, Chinese Academy of Sciences, Beijing 100083, China

**Keywords:** ferroelectric, wurtzite, AlScN

## Abstract

Ferroelectric, phase-change, and magnetic materials are considered promising candidates for advanced memory devices. Under the development dilemma of traditional silicon-based memory devices, ferroelectric materials stand out due to their unique polarization properties and diverse manufacturing techniques. On the occasion of the 100th anniversary of the birth of ferroelectricity, scandium-doped aluminum nitride, which is a different wurtzite structure, was reported to be ferroelectric with a larger coercive, remanent polarization, curie temperature, and a more stable ferroelectric phase. The inherent advantages have attracted widespread attention, promising better performance when used as data storage materials and better meeting the needs of the development of the information age. In this paper, we start from the characteristics and development history of ferroelectric materials, mainly focusing on the characteristics, preparation, and applications in memory devices of ferroelectric wurtzite AlScN. It compares and analyzes the unique advantages of AlScN-based memory devices, aiming to lay a theoretical foundation for the development of advanced memory devices in the future.

## 1. Introduction

Ferroelectric materials have unique characteristics that enable them to maintain their polarization state under zero bias voltage. The switching phenomenon caused by changes in the polarization state when an external bias voltage is applied can be used as a memory device. Such memory devices are fundamentally non-volatile, meaning that they retain stored information even in the absence of power [1]. Compared to traditional silicon-based memory devices such as volatile static random-access memory (SRAM) [2], dynamic random-access memory (DRAM) [3], or non-volatile Flash memory [4] and electrically erasable programmable read-only memory (EEPROM) [5], ferroelectric memory devices exhibit superior performance in terms of faster read and write speeds, higher endurance, and more prolonged data retention capabilities [6,7,8,9]. Ferroelectric materials form the core of ferroelectric memory devices and have been intensively researched. Notably, perovskite structures such as barium titanate (BT) and lead zirconate titanate (PZT), as well as fluorite structures like hafnium zirconium oxide (HZO), have garnered considerable attention. These materials show their advantages in various aspects. For instance, BT exhibits a high dielectric constant [10,11]. PZT combines high dielectric constant, pyroelectric effect, and piezoelectric effect [12,13]. HZO demonstrates the capability to be scaled down to the nanoscale [14,15]. As early as 1992, Ramtron Corporation introduced FM 1208, which is a 4 Kb parallel input/output (I/O) memory device that utilizes PZT as its ferroelectric layer [16].

Although perovskite and fluorite structures of ferroelectric materials show great promise, they still possess inherent limitations. Perovskite structure struggles to break through in terms of size and is incompatible with complementary metal oxide semiconductor (CMOS) processes [17]. Additionally, the inclusion of lead-based toxic materials, such as PZT, poses significant environmental challenges [18,19]. The fluorite structure seems to exhibit high scalability and perfect compatibility with CMOS processes [20]. However, the stability issues arising from polycrystalline and multiphase structures hinder their ability to ensure reliable data storage capabilities after multiple cycles [21,22]. These problems are expected to be resolved in ferroelectric wurtzite aluminum scandium nitride (AlScN), which exhibits significantly higher remanent polarization (*P_r_*) and coercive field (*E_c_*), as well as increased breakdown field and curie temperature (*T_c_*), providing guarantees for improved stability and endurance. It is lead-free and compatible with CMOS processes [23,24], laying the foundation for their commercial applications. The summarized properties of three different structures of ferroelectric materials (including PZT, HZO, and AlScN) are presented in Table 1, indicating that AlScN has advantages in memory window (MW), data retention ability, and temperature reliability due to its larger *E_c_*, *P_r_*, and *T_c_* so that it is more suitable to be the foundation material for advanced memory devices. This is of great significance for advancing the development of artificial intelligence (AI) and adapting to the explosive growth of information in society.

In this work, we summarize the recent research progress in the characteristics and preparation processes of the ferroelectric wurtzite AlScN. Further, we focus on applications in advanced memory devices such as ferroelectric random-access memory (FeRAM), ferroelectric field-effect transistor (FeFET), and ferroelectric tunnel junction (FTJ). Because AlScN-based memory devices are still in the exploratory stage and lack a summary, we have provided a detailed analysis and evaluation from various aspects such as structure and performance. Finally, perspectives are put forward on the current problems of AlScN-based memory devices, and reasonable suggestions are made for their future development, aiming to inspire the development of advanced memory devices.

## 2. Material Properties and Preparation Processes

**Figure 1:**
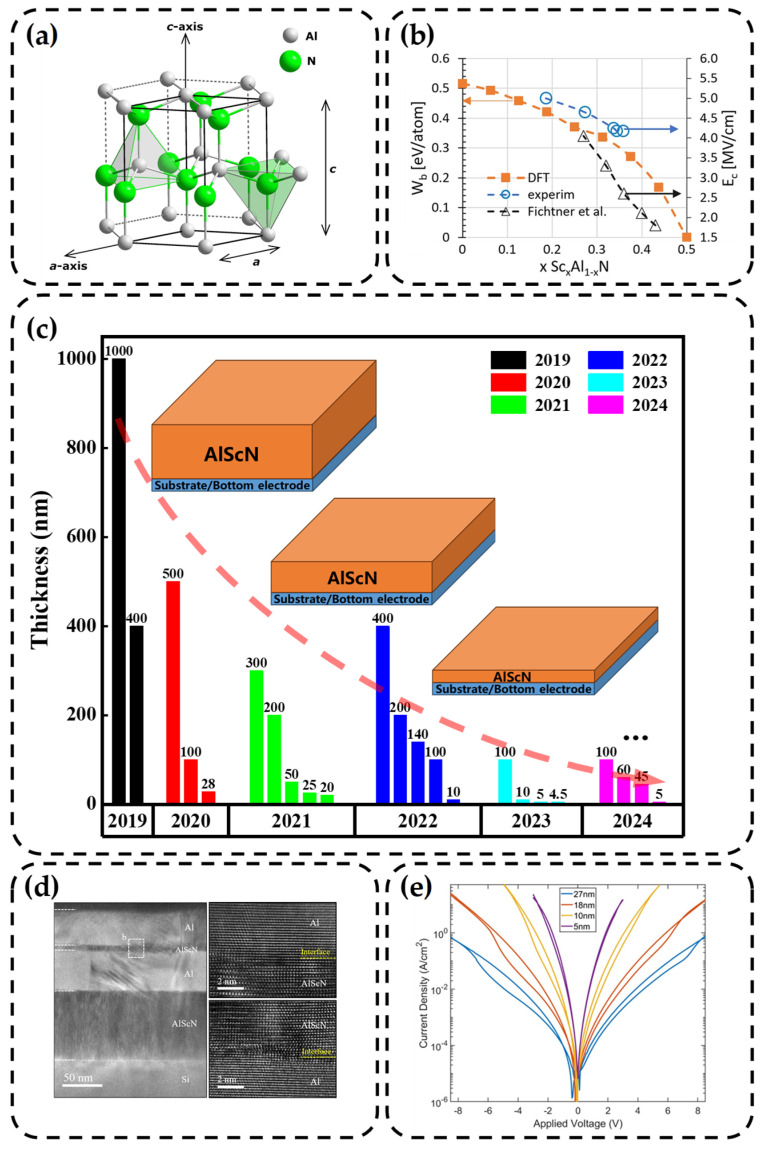
(**a**) Crystal structure of AlN [45]. Copyright 2022, IEEE. (**b**) The influence of Sc components on AlScN polarization [47]. Copyright 2021, AIP Publishing. (**c**) Research trends in AlScN film thickness in recent years [23,34,36,43,48,49,50,51,52,53,54,55,56,57,58,59,60,61,62,63,64,65,66,67,68]. (**d**) The cross-sectional TEM image of Al/AlScN/Al and interfaces between layers [69]. Copyright 2023, AIP Publishing. (**e**) Current density of 27, 18, 10, and 5 nm thick AlScN films as a function of voltage [69]. Copyright 2023, AIP Publishing.

The ferroelectricity of wurtzite AlScN was first reported by Fichtner et al. in 2019 [34]. Thanks to the unique hexagonal close packing of the wurtzite structure [24,40], the strong chemical bonds [41], and the wide bandgap [42], it exhibits high stability and endurance [24,43,44]. AlScN has a subtle relationship with aluminum nitride (AlN). AlN is shown in Figure 1a, which is also a wurtzite structure [45], but in most cases, it does not possess ferroelectricity because polarization reversal cannot occur before the dielectric breakdown, indicating that *E_c_* is too high [34,46].

First-principles calculations have been proven to be an effective means of predicting material properties. Through such calculations, it has been demonstrated that appropriate doping of Sc in AlN can effectively reduce *E_c_* [42,70]. Sc was chosen because it belongs to the group III element, and the most direct way to alter the chemical properties of AlN is by substituting elements from either the III or V group. However, elements such as phosphorus (P) and thallium (Tl) either have low solubility in AlN or possess toxicity, making their practical application challenging [35]. As a transition metal element of Group III, Sc has an oxidation state of +3, an empty 3d orbital, and its atomic size is relatively matched with Al. There have been examples of transition metal elements forming alloys with AlN, such as TiN/AlN, so it is indeed a promising candidate. Research has shown that the incorporation of Sc can effectively reduce the lattice parameter *c/a* (*a* and *c* are lattice constants of hexagonal structure), leading to an increase in the corresponding internal parameter *u* [34,71], as indicated by the following equation:

(1) $$u=\frac{1}{3}\frac{a^{2}}{c^{2}}+\frac{1}{4}$$

Inducing lattice distortion while enhancing the ionic bond [72,73,74], so the energy barrier between polarization directions is reduced [47,75], as shown in Figure 1b. However, it is important to note that a higher proportion of Sc is not always advantageous. There is a critical value for *x* in Al_1−*x*_Sc*_x_*N, which is approximately 0.56 [35,42]. Below this value, the material maintains the wurtzite phase, while exceeding it may result in a rock-salt phase where ferroelectricity may no longer be present [76].

Additionally, the thickness of the film is a matter of great concern due to the huge *E_c_* of AlScN. This translates to a higher operating voltage requirement, posing challenges in meeting the demands of integrated circuit development [48]. Empirically, adjusting *E_c_* can be effectively achieved by altering the thickness of the thin film. So, we have summarized recent research on the thickness of AlScN films [23,34,36,43,48,49,50,51,52,53,54,55,56,57,58,59,60,61,62,63,64,65,66,67,68], as depicted in Figure 1c. Such research of thickness reveals a significant downward trend, while the corresponding Sc components gradually concentrate around 0.3. In 2023, it appears to represent a new milestone, with thickness even dropping below 5 nm. This demonstrates the remarkable scalability of AlScN. Schönweger et al. summarized that with the thickness decreases from 100 nm to 10 nm [48], *E_c_* increases, possibly due to lattice mismatch-induced stress changes. However, when the thickness reduces to below 10 nm, *E_c_* further decreases, which is consistent with Dawber et al.’s theory and is associated with the introduction of depolarization fields [77]. It is worth noting that AlScN will react with oxygen to form an oxide layer if exposed to the air. Fortunately, this process is self-limiting and will not diffuse to the interior of the film. However, once low-density grain boundaries exist, it still leads to oxygen diffusion. The presence of the oxide layer alters the surface composition and microstructure of the film. Particularly, as the proportion of the oxide layer increases at the nanometer scale, it will have a significant impact on the performance [78].

In addition to various fascinating characteristics, multiple fabrication processes for AlScN thin films have been proposed. Through our research, we have identified the main utilization of the following three methods: physical vapor deposition (PVD), molecular beam epitaxy (MBE), and metal-organic chemical vapor deposition (MOCVD). Next, we will describe each method based on the reported experimental results.

### 2.1. Physical Vapor Deposition

PVD is widely used due to its simple mechanism. It only involves physical changes, and the target atoms are deposited on the substrate by bombarding with argon ions in the vacuum environment, which can be used for metals, semiconductors, insulators, and other materials. During the process, some reaction gases or substrate heating can also be introduced to meet special requirements.

As early as 2009, Akiyama et al. prepared AlScN alloy thin films by dual reactive co-sputtering [74]. They achieved the desired Sc concentration by adjusting the sputtering power of Al and Sc targets on an *n*-type (100) silicon substrate at a relatively low temperature of 580 °C. The atmosphere during deposition consisted of nitrogen (99.999%) and argon (99.999%) with a ratio of 4:6. This method allows for precise control of the Sc concentration. However, since it involves two target materials, the preparation time is longer. Subsequently, researchers like Rassay attempted to combine Al and Sc on a single target through target design [64]. They successfully achieved single-alloy target sputtering, which offers a higher deposition rate, making it advantageous for industrial production. However, due to the already designed target, further adjustment of the Sc concentration is not possible, reducing its flexibility.

So far, the sputtering method of AlScN has successfully entered the 5 nm thick technology node; the lattice matching and the impact of the oxide layer are very fatal. Zheng et al. strictly controlled the sputtering environment and successfully deposited Al/AlScN/Al films without damaging the vacuum [79], as shown in Figure 1d. The clear interface between Al and AlScN indicates the absence of an oxide layer. Furthermore, they successfully observed the hysteresis in the current–voltage (*I*–*V*) curves using a quasi-DC measurement method, as shown in Figure 1e. This effectively validates the ferroelectric switching behavior present in AlScN thin films prepared through sputtering.

**Figure 2:**
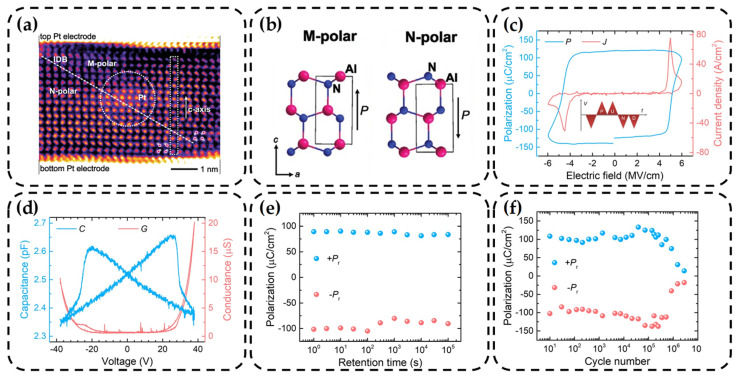
(**a**) Inverted-ABF-STEM micrograph of the full Al_0.74_Sc_0.26_N layer featuring an inclined inversion domain boundary separating regions of M-polarity (upper right) and N-polarity (lower left) [48]. Copyright 2023, Wiley-VCH. (**b**) Sketches of the atomic structure in the M- and N-polar state along the line of sight [48]. Copyright 2023, Wiley-VCH. (**c**) *J–E* and *P–E* loops from a triangular waveform PUND measurement [80]. Copyright 2022, AIP Publishing. (**d**) Butterfly-shaped *C–V* loop and the corresponding conductance recorded on a device with a top electrode diameter of 50 μm at 1 MHz (AC = 100 mV) [80]. Copyright 2022, AIP Publishing. (**e**) Retention and (**f**) endurance test for ferroelectric N-polar AlScN [80]. Copyright 2022, AIP Publishing.

Likewise, Schönweger et al. achieved the sub-5 nm AlScN films on silicon substrates through sputtering. A platinum (Pt)/AlScN/Pt structure was employed to achieve ferroelectric switching below 1 V [48]. They successfully observed nitrogen (N)-polar and metal (M)-polar regions within individual grains using annular bright-field scanning transmission electron microscopy (ABF-STEM), as shown in Figure 2a. This observation serves as compelling evidence for the existence of inversion domain boundaries (IDB). Their research represents a significant breakthrough, indicating that ferroelectric wurtzite AlScN can be deposited on conventional silicon substrates using sputtering methods that are compatible with CMOS processes. Moreover, it can operate at voltages as low as 1 V, thereby paving the way for the integration of AlScN-based ferroelectric devices with other parts.

### 2.2. Molecular Beam Epitaxy

MBE is a specialized thin film deposition technique that involves heating individual component elements to vaporize them and then growing them on the substrate in the form of molecular beams. This process enables the production of thin films with superior crystal quality, reduced roughness, and directional growth characteristics. In comparison to sputtering methods of AlScN, MBE holds the promise of achieving lower impurity levels and higher purity wurtzite phase thin films, which is highly advantageous for preserving the ferroelectric properties of AlScN.

In 2017, Hardy et al. first reported the growth of 200-nm-thick AlScN thin films with Sc ratios ranging from 0.14 to 0.24 on gallium nitride (GaN) and silicon carbide (SiC) substrates using MBE [79]. The growth temperature ranged from 360 °C to 890 °C. They further investigated metal-rich and nitrogen-rich growth conditions by adjusting the flux of III/V elements. The results indicated a slight decrease in phase purity for metal-rich growth, possibly due to the excessive reaction between Sc and Al. The root mean square (rms) roughness of the thin films was as low as 0.7 nm, and the rocking curve with a full width at half maximum as low as 265 arcsecs.

In 2020, Casamento et al. further investigated the surface roughness of AlScN thin films within the Sc ratio range of 0.18~0.40. They explored different III/V ratios and substrate temperatures; the thickness of the films is ~28 nm [51]. Their findings were consistent with Hardy’s conclusions, showing that films grown under metal-rich conditions exhibited significantly higher roughness. Notably, a smoother surface was achieved at a substrate temperature of 600 °C under nitrogen-rich growth conditions. This was attributed to the absence of Al desorption and the formation of the ScN phase at this temperature. In the same year, Wang et al. also successfully grew pure wurtzite phase AlScN thin films with a Sc ratio as high as 0.34 using MBE. These films exhibited rms roughness of less than 1 nm. Remarkably, AlScN, with a Sc ratio of 0.2, maintained its wurtzite structure at high temperatures up to 900 °C [53], indicating the inherent thermal stability of AlScN. This suggests the potential application of AlScN in demanding high-temperature environments.

Indeed, epitaxially grown AlScN exhibits both M-polar and N-polar orientations, as illustrated in Figure 2b [48], each possessing distinct properties. Wang et al., in their 2021 study, demonstrated that M-polar AlScN is poised to face scaling challenges, whereas N-polar AlScN, characterized by lower sheet resistance and other favorable attributes, exhibits inherent scaling advantages. Furthermore, they established that the polarity of AlScN is closely linked to the substrate [81], a finding of profound significance for subsequent research into AlScN-based devices. This underscores the imperative of meticulous consideration of lattice matching between the substrate and the thin film.

In 2022, Wang et al. further demonstrated the ferroelectricity of single-crystalline wurtzite phase N-polar AlScN with a thickness of 100 nm grown on sapphire substrates using the MBE method. To mitigate the influence of leakage currents, a triangular waveform positive-up-negative down (PUND) bias sequence with a frequency of 10 kHz was employed for testing [80]. The obtained current density–electric field (*J–E*) and polarization–electric field (*P–E*) results, as shown in Figure 2c, along with the clear butterfly-shaped capacitance–voltage (*C–V*) curve presented in Figure 2d, indicate that the prepared AlScN possesses a significant *E_c_* and *P_r_*. Furthermore, retention and endurance tests, as depicted in Figure 2e,f, demonstrate a retention time of up to 10^5^ s and over 5 × 10^5^ switching cycles. It can be seen that the ferroelectric wurtzite AlScN film grown by MBE can obtain better quality and can be used as a means to optimize performance.

### 2.3. Metal Organic Chemical Vapor Deposition

MOCVD is the preferred method for industrial production of nitride semiconductors such as GaN and AlN [82,83]. It involves depositing films on a substrate by causing a chemical reaction between precursors and reaction gases under controlled parameters such as flow rate, temperature, and pressure. MOCVD offers high growth rates and is widely used due to its ability to precisely control growth conditions. The key factors in MOCVD are the selection of suitable precursors and the control of growth parameters. The method for preparing ferroelectric wurtzite AlScN thin films using MOCVD has generally achieved consensus regarding the introduction of Al element and the choice of reaction gases, namely trimethylaluminum (TMAl) and ammonia (NH_3_). However, difficulties arise when selecting the precursor for Sc, which is also one of the reasons why this method has not been extensively reported. Choices for Sc precursors mainly include tris(methylcyclopentadienyl)scandium (MeCp_3_Sc), tris(cyclopentadienyl)scandium (Cp_3_-Sc), and bis(methylcyclopentadienyl)scandium chloride ((MCp)_2_ScCl). MeCp_3_Sc has a rather complex synthesis route and exhibits a low vapor pressure, which is unfavorable for both scientific research and commercial production. Cp_3_Sc, on the other hand, is a commercially available precursor and has been more extensively documented.

In 2020, Leone et al. conducted a detailed investigation into the reaction conditions of Cp_3_Sc and found that a temperature as high as 150 °C is required to achieve a sufficient molar flow [84]. However, this temperature poses a significant challenge for MOCVD equipment because various components responsible for controlling the flow include sealing materials and electronic parts that are not well-suited to withstand such high temperatures. Nevertheless, through the diligent efforts of Leone et al., these challenges were successfully overcome, leading to the synthesis of AlScN films with Sc content as high as 30% and exhibiting superior crystalline quality. This represents a significant breakthrough because prior research, such as that by Saidi and others, had primarily focused on lower levels of Sc doping using Cp_3_Sc [85]. In the 2021 report by Manz et al., it was mentioned that in the MOCVD method, Al atoms tend to diffuse into the nitride substrate at high temperatures. They also employed TMAl and Cp_3_Sc and observed that the vertical distribution of Al and Sc in heterostructures was nonuniform [86]. This represents an area in MOCVD that requires further optimization.

In response to the vapor pressure issue with the Sc precursor, Streicher et al. proposed the use of (MCp)_2_ScCl in 2023. Its vapor pressure is more than ten times higher than that of Cp_3_Sc, enabling an increase in the molar flow rate and consequently enhancing the growth rate. Importantly, Cp_3_Sc contains 15 carbon atoms for each Sc atom, whereas (MCp)_2_ScCl only has 12 carbon atoms (with no adverse effects from chlorine atoms on the growth layer) [87]. This suggests that (MCp)_2_ScCl precursor introduces fewer impurities, which is crucial for ensuring the purity of AlScN.

## 3. Applications in Advanced Memory Devices

**Figure 3:**
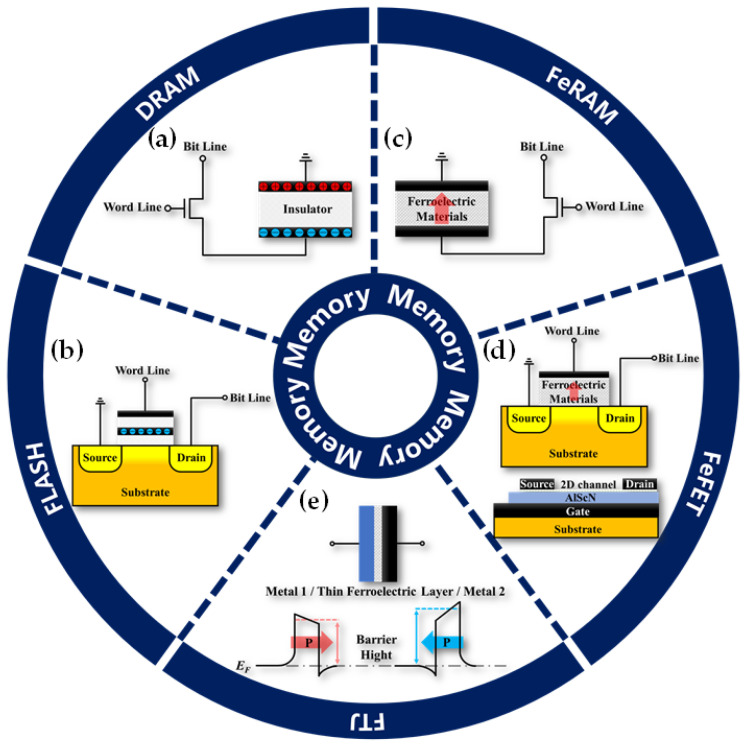
(**a**) Typical structure of 1T-1C DRAM. (**b**) Basic unit structure of Flash memory. (**c**) Typical structure of 1T-1C FeRAM. (**d**) Two different structures of FeFET. The structure on the upper side is similar to a floating gate transistor, while for the lower structure, the gate is on the lower substrate, and 2D material is used as the channel. (**e**) Schematic diagram of FTJ structure and barrier height determined by polarization state.

Since the invention of the computer, memory devices have become one of the most crucial electronic components. DRAM for temporary data storage and Flash memory for long-term data storage play exceedingly significant roles in storage architectures. The classic structure of DRAM, as shown in Figure 3a, is composed of a transistor and a capacitor in series. The gate and drain of the transistor are connected, respectively, to the word line and the bit line. The values 0 and 1 are defined by the presence or absence of charge in the capacitor, and together, they enable read, write, and erase operations for each bit. Table 2 lists the technical parameters that DRAM has achieved in recent years, demonstrating its unique advantages, such as ultra-high write speed and lower operating voltage. The basic unit of Flash memory is a floating gate transistor shown in Figure 3b, which is very similar to the structure of a metal oxide semiconductor field effect transistor (MOSFET), but there is an additional floating gate in the insulation layer, which can store or release charges in combination with word line and bit line, thereby representing 1 and 0. Moreover, due to the encapsulation of insulators around the floating gate, the stored charges are not easily escaped, thus achieving long-term information preservation even when power is cut off. After adopting different connection methods for floating gate transistors, they developed into NAND Flash and NOR Flash. Table 2 shows the overall performance of Flash memory in recent years, demonstrating attractive long data retention and low power consumption.

Now, with the continuous development of the Internet of Things (IoT) and AI, demand for data storage has grown exponentially, creating a vast market. However, at the same time, the requirements for memory devices have also increased, including the need for higher storage density, better endurance, and lower power consumption [104]. However, efforts such as 3D stacking have been made to improve the performance of traditional silicon-based memory devices like DRAM and Flash memory [105]. However, they still face inherent limitations. DRAM, for example, is susceptible to data loss after power interruption and tends to have relatively high-power consumption. Flash memory, on the other hand, is limited by a finite number of erase/write cycles and can be sensitive to high temperatures [106]. Indeed, innovating at the level of device materials and structures is often considered the most effective way to improve the performance of memory devices. This viewpoint has been proven in a considerable amount of research on ferroelectric memory devices such as FeRAM and FeFET, which are based on PZT [107] or HZO [108].

Ferroelectric memory devices are regarded as advanced memory devices due to their high read and write speed, high durability, low power consumption, non-volatility, and good data retention. This requires good ferroelectricity of ferroelectrics as support. For example, FeRAM requires as much *P_r_* and stable domains as possible to ensure bit density and retention time. FeFET needs a slightly larger *E_c_* to ensure MW and resist interference. FTJ requires a sufficiently large *E_c_* and *P_r_* to maintain a high MW and ON/OFF ratio when scaled down to the thickness of several nanometers. However, due to some genetic limitations such as complex processes, poor compatibility, depolarization effects, fatigue effects, and difficulty in size reduction, it has been difficult to achieve large-scale applications.

As a promising ferroelectric wurtzite material since the 100th anniversary of the discovery of ferroelectricity, AlScN itself has larger *E_c_* and *P_r_*, better temperature characteristics, and a more stable phase, which is expected to overcome the long-term genetic defects of ferroelectric memory materials. It is even believed to have the potential to change modern microelectronics [109]. Therefore, we summarize various AlScN-based memory devices reported in recent years and analyze the application prospects in advanced memory devices.

### 3.1. Ferroelectric Random Access Memory

The structure of FeRAM is highly similar to DRAM, as shown in Figure 3c. The key distinction lies in replacing the insulator in DRAM with a ferroelectric layer. From a manufacturing perspective, if the ferroelectric materials in FeRAM can be compatible with current CMOS processes, FeRAM is more likely to be mass-produced. Furthermore, an applied electric field induces polarization of the ferroelectric material in a specific direction, which can be utilized to represent data in different polarization states. Because these polarization states remain unchanged even when the external electric field is removed, FeRAM exhibits non-volatile characteristics. This is a highly competitive advantage, as it overcomes the drawback of DRAM, which requires frequent refreshing, significantly reducing power consumption during usage [110]. When combined with the excellent inherent properties of the material itself, these advantages become even more intriguing. The previous discussion has thoroughly analyzed the properties of the ferroelectric wurtzite AlScN, and it can be predicted that AlScN-based FeRAM must have unique properties.

The key to achieving low power consumption, high speed, endurance, and good retention characteristics in FeRAM lies in the ferroelectric capacitors. In recent years, there have been two main types of AlScN-based ferroelectric capacitors: metal-ferroelectric-metal (MFM) and metal-ferroelectric-semiconductor (MFS). Gund et al. provided a structural discussion and reported vertical and lateral MFM-structured AlScN-based ferroelectric capacitors in 2021. Molybdenum (Mo) and Al were used as the bottom electrode and top electrode, respectively [54]. The results indicated that the lateral structure capacitor appeared to have a smaller leakage current, and they attributed this to the number of barrier crossings by the current. Leakage current is a challenging issue inherent to AlScN, and Gund’s findings suggest that manufacturing ferroelectric capacitors in a lateral structure could be explored as a means to reduce the impact of leakage current in FeRAM.

**Figure 4:**
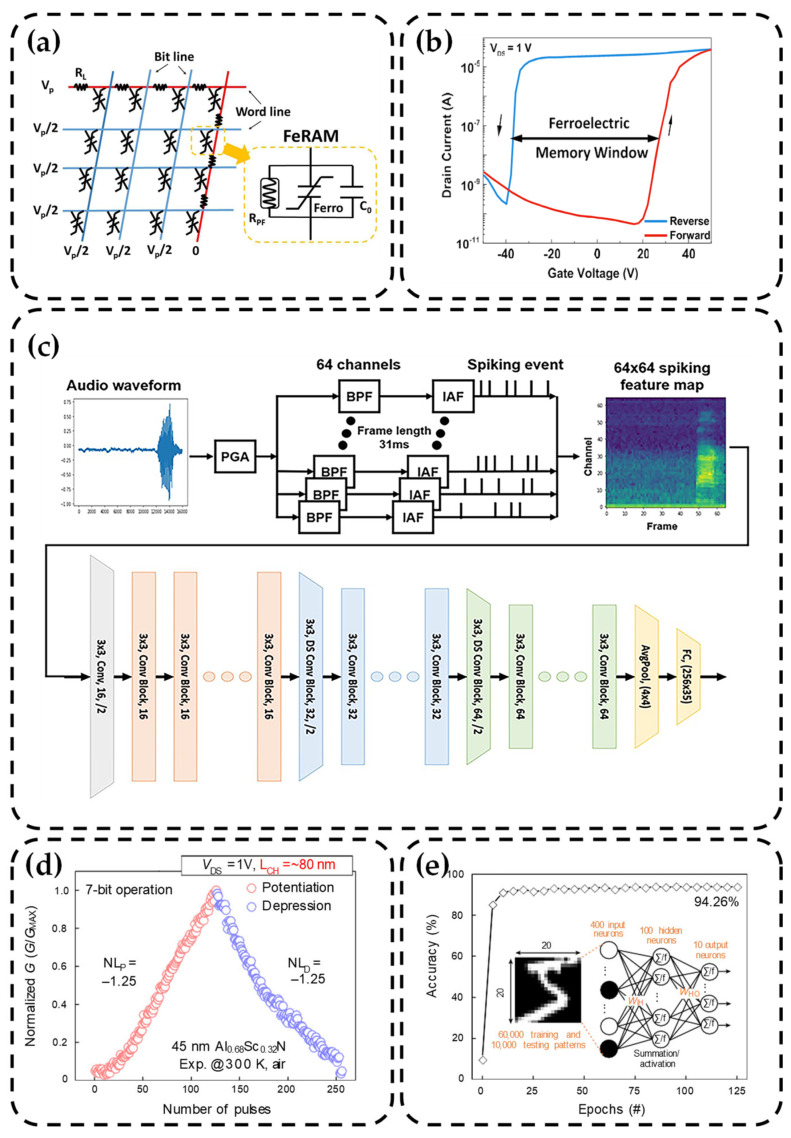
(**a**) Diagram of the selector-free array and the circuit model of the FeRAM cell [111]. Copyright 2021, IEEE. (**b**) Room temperature semi-logarithmic scale transfer characteristics of typical AlScN/MoS_2_ FE-FET [113]. Copyright 2021, American Chemical Society. (**c**) The diagram of the speech recognition system with feature extraction module and ResNet implemented by FeRAM crossbar array [114]. Copyright 2023, IEEE. (**d**) 7-bit conductivity state of pulse programming operation [115]. Copyright 2023, Springer Nature. (**e**) A schematic of an MLP-based ANN with a size of 400 × 100 × 10 [115]. Copyright 2023, Springer Nature.

Liu et al. researched the issue of leakage current in AlScN. They prepared MFM-structured capacitors with Mo as the bottom electrode and Pt as the top electrode [111]. Through a combination of experimental data and calculations, they discovered that the leakage current is primarily driven by electron emission and hopping assisted by nitrogen vacancies. This behavior aligns with the Poole–Frenkel (P–F) emission mechanism. As a result, they suggested that depositing AlScN films in an N-rich environment could be considered to reduce leakage current. Additionally, they constructed a non-selective array of AlScN-based FeRAM, as shown in Figure 4a. Each unit comprises three parts: R_PF_ to describe the leakage component, the middle part to characterize the ferroelectric behavior, and *C_0_* representing the experimentally measured capacitance of 0.15 nF/cm^2^. Through simulations at different frequencies, they found that the non-selective FeRAM array suffers from delay and read interference issues. The delay is primarily related to capacitance (*τ* = *RC*), while interference is mainly associated with leakage current. Wang et al. analyzed the ferroelectric switching characteristics at high frequencies from the perspective of domain wall motion [112]. They pointed out that domain wall motion in AlScN does not introduce significant delays, their capacitors achieved a ferroelectric switching time of 200 ns, and *P_r_* did not decrease after 8.7 × 10^3^ cycles. Therefore, future designs of AlScN-based FeRAM arrays should consider reducing the unit area and optimizing the process environment to enhance performance.

### 3.2. Ferroelectric Field Effect Transistor

The classical metal-ferroelectric-insulator-semiconductor (MFIS) structure of FeFET, as shown on the upper side of Figure 3d, is similar to the basic unit of Flash memory. However, instead of relying on a floating gate, it utilizes the polarization of the ferroelectric thin film to control the channel between the source and drain, effectively adjusting the threshold voltage (*V_th_*) and achieving non-volatile data storage. Due to the presence of regularly aligned dipoles in the polarized ferroelectric layer, a uniform induced electric field can be generated to regulate the channel. Therefore, FeFET often requires only a single gate. Reading operations only require applying a small voltage on the word line to obtain current information on the bit line, which does not significantly affect the polarization state of the ferroelectric layer. Consequently, FeFET not only addresses the high operating voltage issue associated with the tunneling mechanism in Flash memory but also resolves the destructive read problem in FeRAM, resulting in lower power consumption. This kind of structure is often seen in HZO-based FeFET [116].

For AlScN-based FeFET devices, the structure commonly adopted is as depicted on the lower side of Figure 3d. The gate is situated at the bottom and can be either doped semiconductor or metal. Above the gate, there is a ferroelectric thin film, and the source and drain are determined by defined metal patterns, with a two-dimensional material serving as the channel layer. Given the significant impact of lattice matching and interface effects on the ferroelectric properties of AlScN, this design is particularly ingenious. When combined with high *P_r_* and *E_c_* of AlScN, it can achieve longer data retention and larger MW, making it a highly promising candidate for the basic unit of advanced memory devices.

A brief comparison with other FeFET devices based on 2D materials/ferroelectrics is shown in Table 3. The larger *E_c_* of AlScN provides a significantly better MW compared to HZO and PZT, which can greatly prevent noise interference and be crucial for the reliability of data storage. Additionally, it effectively resists the depolarization field and achieves longer retention times. Although it appears that excessive working voltage may cause poor endurance, this is not fatal. AlScN can completely reduce the working voltage by changing its composition or thickness. A larger *E_c_* can help maintain MW at smaller features, which also supports improving storage density.

In 2021, Liu et al. combined two-dimensional (2D) molybdenum disulfide (MoS_2_) with AlScN to design FeFET, which is a bottom-gated transistor and compatible with the back end of the line (BEOL) processes. A 100 nm AlScN film was deposited on Pt by sputtering, and MoS_2_ was transferred using a mechanical exfoliation method with tape, ensuring high quality and accurately reflecting the performance of AlScN. Due to the presence of sulfur vacancies in the channel layer, FeFET exhibited an n-type behavior. Electrical testing yielded a counterclockwise hysteresis loop, as shown in Figure 4b, with a remarkable normalized MW of up to 0.3 V/nm, effectively preventing random switching errors and read interference. It also exhibited stable performance with a large ON/OFF ratio of 10^6^ over 10^4^ cycles and a retention time of up to 10^5^ s. Additionally, they demonstrated a switching speed of <200 ns. It is evident that in terms of cycling endurance, AlScN-based FeFETs have reached a level comparable to Flash memory but with significantly higher speed [113]. The innovative approach of Liu et al. indicates that the integration of a two-dimensional channel with AlScN in FeFETs effectively optimizes data retention times, making it an ideal choice for embedded memory and memory-based computing architectures. It also opens up new opportunities for direct memory integration with logic transistors.

In 2023, Kim et al. achieved significant advancements in FeFET by combining a monolayer MoS_2_ (~0.7 nm) with AlScN. The 45 nm and 100 nm AlScN films were deposited by co-sputtering, and the substrate temperature was maintained at 350 °C during deposition. MoS_2_ was deposited on the sapphire by CVD and MOCVD, then transferred using the wet transfer method. Their work can be summarized in three key aspects [115]. (1) By reducing the thickness of AlScN and increasing the Sc content, they were able to control the MW and *V_th_*. This adjustment allowed AlScN-based FeFET to operate at lower voltages compared to Flash memory, making them suitable for low-power integration with Si CMOS technology and monolithic 3D integration. (2) The devices featured a channel length as small as 78 nm, combined with an impressive ON/OFF current ratio of 10^7^ and a current density of 252 μA/μm. This outstanding scalability was attributed to the strong electrostatic control of MoS_2_ and the high *P_r_* of AlScN, which offered valuable insights into achieving high current density and mitigating short-channel effects in high-speed devices. (3) The AlScN-based FeFET demonstrated a remarkable 10-year data retention capability and endurance exceeding 10^4^ cycles. Moreover, they were employed as artificial synapses with 7-bit operation. These devices can also exhibit data retention and endurance comparable to Flash memory in circuit applications, based on pulsed programming and erasing of the resistance states. The ability to achieve multiple memory states within a single cell may be attributed to the presence of multiple ferroelectric domains beneath the channel, resulting in local ferroelectric switching. So, researchers successfully demonstrated 7-bit conductance states for pulse programming of artificial synapses, as evidenced by the update of synaptic weights in Figure 4d. Furthermore, an artificial neural network (ANN) simulation based on multi-layer perceptron (MLP) was conducted using open-source code NeuroSimV3.0, as shown in Figure 4e, with a maximum accuracy of 94.26%.

Just as MOSFETs can be classified into n-channel and p-channel based on their conduction types, FeFETs follow a similar categorization. Kim et al. further replaced MoS_2_ with 2D material tungsten diselenide (WSe_2_) as a channel material in 2024 [66], successfully realizing p-type FeFETs, attributed to the presence of numerous W vacancies in WSe_2_ [120]. Compared to *n*-type FeFETs, *p*-type FeFETs exhibit hysteresis loops in the opposite direction. They also pointed out that metal for the source and drain electrodes plays a crucial role in tuning FeFET behavior. Due to variations in the work functions of different metals, there are significant differences in the position of the Fermi level (*E_F_*) in the energy band when in contact with the channel layer. Metals with higher work functions, such as Pt, palladium (Pd), and aurum (Au), tend to have an *E_F_* closer to the valence band, making them suitable for *p*-type applications. Conversely, metals with lower work functions, such as indium (In) and titanium (Ti), typically have an *E_F_* closer to the conduction band, making them suitable for *n*-type applications.

However, it is essential to consider the thickness of 2D materials because single-layer and multi-layer may exhibit significant differences in electron affinity and bandgap [121,122]. Integrated with AlScN and WSe_2_, a high ON/OFF ratio exceeding ~10^7^ and endurance cycles of >5000 have been achieved. This indicates that undoped FeFET technology using 2D materials combined with AlScN opens the door to the development of advanced memory devices and neural mimicry chips.

### 3.3. Ferroelectric Tunnel Junction

In Figure 3e, FTJ is a type of MFM structured device featuring an ultra-thin ferroelectric layer. As the thickness of the ferroelectric layer reduces to sub-20 nm, the quantum mechanical tunneling effect significantly influences the electrical characteristics. Consequently, the barrier height between the ferroelectric layer and metals becomes a critical factor, primarily determined by the polarization. Under the influence of an external electric field greater than *E_c_*, the polarization state can achieve non-volatile switching, resulting in significantly different tunneling currents. This variation is described by the tunneling electroresistance (TER). Its state can be captured by applying a small electric field not exceeding *E_c_*, enabling FTJ to achieve a non-destructive read [23]. FTJs based on HZO often require additional insertion layers to ensure a high TER ratio [123]. Thanks to the large *E_c_* and *P_r_* of AlScN, AlScN-based FTJ has a very high ON/OFF ratio and can maintain a certain state for a long time. This makes them a promising candidate for an outstanding non-volatile memory device [124].

**Figure 5:**
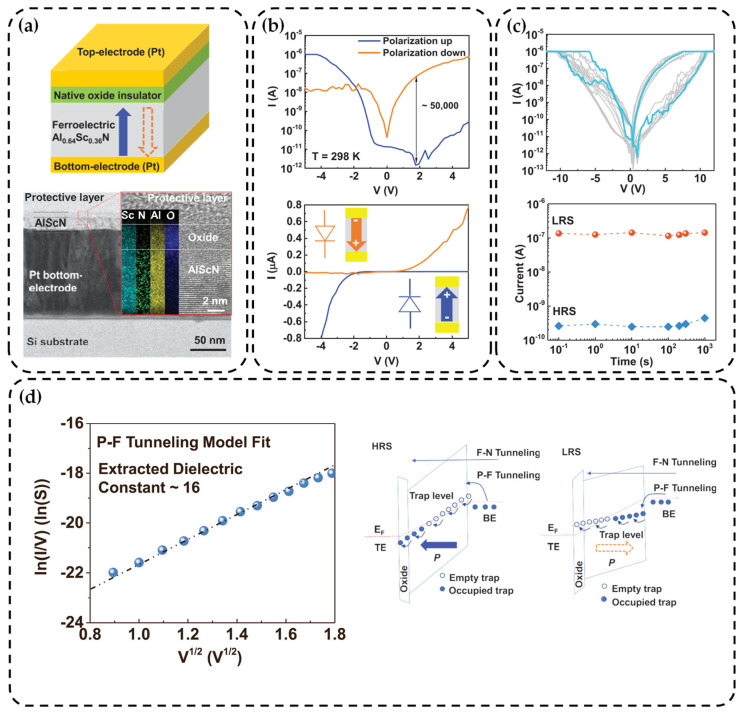
(**a**) AlScN-based FTJ and the combination diagram of cross-sectional transmission electron microscopy and Energy Dispersive Spectrometer (EDS) containing a thin oxide layer [125]. Copyright 2021, AIP Publishing. (**b**) The *I–V* curve with rectification characteristics has a semi-logarithmic curve on the left, with a rectification ratio of about 50,000. The right side shows the relationship between the polarization direction and the current direction [125]. Copyright 2021, AIP Publishing. (**c**) The left side shows the I–V characteristic curve obtained from 10 cycles, which exhibits resistance-switching characteristics. The right side displays the retention time of high and low resistance exceeding 1000 s [125]. Copyright 2021, AIP Publishing. (**d**) The defect-induced electronic band diagrams of HRS and LRS and fitting of experimental results (The blue ball is the experimental result and the line is the fitting result), ln (*I/V*)∝*V*^1/2^ conforms to the P–F tunneling model [125]. Copyright 2021, AIP Publishing.

In 2021, Liu et al. employed a co-sputtering technique to grow a 20 nm thick AlScN film on a 4-inch silicon substrate [125]. The Sc component was measured at 0.36, and Pt was used for the electrodes. Beneath the top electrode, there is a natural oxide layer, as shown in Figure 5a. The deposition temperature does not exceed 350 °C, making it fully compatible with BEOL processing. With an MFM structure, FTJ exhibits *E_c_* of 6.5 MV/cm and *P_r_* of 25 μC/cm^2^. Additionally, as depicted in Figure 5b,c, it simultaneously demonstrates diode-like rectifying characteristics (rectification ratio > 10^5^) and the ability for resistive switching (ON/OFF ratio ~50,000). This is highly advantageous for constructing crossbar arrays because its self-selecting behavior eliminates the need for additional transistors or selectors to address the issue of crosstalk. Furthermore, they also reported the stable programmability of the device under direct current cycling, demonstrating retention times exceeding 1000 s at 300 K. Due to the presence of numerous point and line defects in the sputtering process for the ferroelectric layer, there are inevitably some trap states within the bandgap. The conduction mechanism is attributed to the P–F tunneling model. This conclusion was supported by fitting the relationship between forward current and applied voltage, As shown in Figure 5d. To investigate the impact of the natural oxide layer, they also measured in-situ deposited FTJ based on AlScN. The results indicated that the oxide layer is not necessary or crucial for demonstrating the ferroelectric effect. Therefore, future efforts should focus on reducing the thickness of AlScN and adopting in-situ growth methods to avoid the oxide layer. This approach holds the potential to achieve higher density and non-destructive readout in advanced memory devices.

## 4. Conclusions and Perspectives

In conclusion, we have summarized the research progress of the ferroelectric wurtzite AlScN that appeared on the 100th anniversary of the discovery of ferroelectricity and conducted an in-depth analysis to understand the origin of its ferroelectric properties. In comparison to perovskite and fluorite structures ferroelectrics, AlScN exhibits a broader range of key properties such as *E_c_*, *P_r_*, and *T_c_*. Additionally, the non-toxic, environmentally friendly, and excellent compatibility with CMOS technology demonstrated by AlScN is highly inspiring, indicating that AlScN is very suitable for large-scale production, paving a promising path for its commercialization. In recent years, various fabrication techniques, including PVD, MBE, and MOCVD, have been employed to prepare thin films of AlScN, with film thickness scaling down from an initial 1 μm to sub-5 nm. Remarkably, the maintained favorable ferroelectricity indicates significant potential for dimensional miniaturization, aligning well with the current trends in integrated circuit (IC) processing. Advanced memory devices such as AlScN-based FeRAM, FeFET, and FTJ have been explored, and their performance in some ways even surpasses traditional memory devices like DRAM and Flash memory that have been in development for decades. These advancements hold promise as alternative solutions to further meet future demands for high storage density and low power consumption. Furthermore, AlScN-based memory devices acting as artificial synapses exhibit high precision in running neural network algorithms for image recognition, which suggests significant potential in integrating storage and computation and inspiring breakthroughs in traditional von Neumann architecture. More detailed exploration in this area is worth exploring in the future.

However, before facing large-scale production for memory devices, challenges associated with ferroelectric wurtzite AlScN still need addressing, such as leakage currents induced by defects, elevated operating voltages, and surface oxidation. Future advancements may involve refining the fabrication process, packaging, and potentially utilizing more precise such as atomic layer deposition (ALD) techniques to reduce defects, decrease thickness, and enhance uniformity. Simultaneously, ferroelectric wurtzite AlScN, as a wide-bandgap material, exhibits a certain lattice compatibility with third-generation wide-bandgap semiconductors. This opens the door for further exploration to address the challenges of traditional memory devices failing in extreme environments, such as high temperatures. We believe that ferroelectric wurtzite AlScN will prove to be a multifaceted material with enormous prospects.

## Figures and Tables

**Table 1:** Characteristics of three different structural ferroelectric materials.

Ferroelectric Materials	PZT	HZO	AlScN
Crystal structure	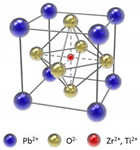	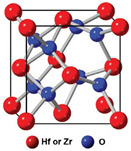	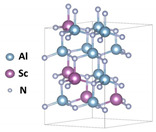
*E_c_* (kV/cm)	~40–80	~1000–2000	~2000–5000
*P_r_* (μC/cm^2^)	~10–40	~15–40	~80–150
*E_g_* (eV)	~2.0–3.8	~5.7–6.5	~2.9–6.1
*Tc* (°C)	~350	~450	>1100
Ref.	[25,26,27,28]	[25,29,30,31]	[32,33,34,35,36,37,38,39]

Copyright 2023, American Chemical Society; Copyright 2023, Wiley-VCH; Copyright 2022, American Chemical Society.

**Table 2:** Recent parameters of traditional memory devices.

	DRAM	Flash Memory
Technology node (nm)	<20	<20
Programming voltage (V)	<2	>10
Endurance (cycles)	>10^16^	<10^5^
Retention	volatile	>10 years
Energy consumption (/bit)	<10 pJ	<1 nJ
Write speed (ns)	<5	>100
Operation temperature (°C)	<85	<85
Ref.	[88,89,90,91,92,93,94,95]	[96,97,98,99,100,101,102,103]

**Table 3:** Parameters of FeFET devices based on 2D materials/ferroelectrics.

FeFET	Graphene/PZT	SnS_2_/HZO	MoS_2_/HZO	MoS_2_/AlScN	WSe_2_/AlScN
MW (V)		>2.5	0.3	~35	>6
ON/OFF ratio	>5	>10^5^	>10^7^	~10^6^	~10^7^
Working voltage (V)	±6		±5.5		±10
Endurance (cycles)		10^7^	10^3^	10^4^	>5000
Retention (s)	>10^4^	10^5^	10^4^	10^5^	10^4^
Ref.	[117]	[118]	[119]	[113]	[66]

## Data Availability

The data that support the findings of this study are available from the corresponding authors upon reasonable request.
